# Influenza A(H1N1)pdm09 Virus Aggravates Pathology of Blood Vessels in Wistar Rats with Premorbid Acute Cardiomyopathy

**DOI:** 10.3390/v15051114

**Published:** 2023-05-04

**Authors:** Vladimir Marchenko, Irina Zelinskaya, Yana Toropova, Ekaterina Podyacheva, Mikhail Martynov, Daria Mukhametdinova, Dmitry Lioznov, Irina N. Zhilinskaya

**Affiliations:** 1 Smorodintsev Research Institute of Influenza, Russian Ministry of Health, 197376 St. Petersburg, Russia; 2 Smorodintsev Research Institute of Influenza, Department of Medical Microbiology, North-Western State Medical University Named after I.I. Mechnikov, 191015 St. Petersburg, Russia; 3 Almazov National Medical Research Centre, Russian Ministry of Health, 197341 St. Petersburg, Russia

**Keywords:** Wistar rats, influenza A(H1N1)pdm09 virus, endothelial factors, mesenteric blood vessels, wire myography, immunohistochemistry, ELISA

## Abstract

Influenza virus can infect the vascular endothelium and cause endothelial dysfunction. Persons at higher risk for severe influenza are patients with acute and chronic cardiovascular disorders; however, the mechanism of influenza-induced cardiovascular system alteration remains not fully understood. The aim of the study was to assess the functional activity of mesenteric blood vessels of Wistar rats with premorbid acute cardiomyopathy infected with Influenza A(H1N1)pdm09 virus. For this, we determined (1) the vasomotor activity of mesenteric blood vessels of Wistar rats using wire myography, (2) the level of expression of three endothelial factors: endothelial nitric oxide synthase (eNOS), plasminogen activator inhibitor-1 (PAI-1), and tissue plasminogen activator (tPA) in the endothelium of mesenteric blood vessels using immunohistochemistry, and (3) the concentration of PAI-1 and tPA in the blood plasma using ELISA. Acute cardiomyopathy in animals was induced by doxorubicin (DOX) following infection with rat-adapted Influenza A(H1N1)pdm09 virus. The functional activity of mesenteric blood vessels was analyzed at 24 and 96 h post infection (hpi). Thus, the maximal response of mesenteric arteries to both vasoconstrictor and vasodilator at 24 and 96 hpi was significantly decreased compared with control. Expression of eNOS in the mesenteric vascular endothelium was modulated at 24 and 96 hpi. PAI-1 expression increased 3.47-fold at 96 hpi, while the concentration of PAI-1 in the blood plasma increased 6.43-fold at 24 hpi compared with control. The tPA concentration in plasma was also modulated at 24 hpi and 96 hpi. The obtained data indicate that influenza A(H1N1)pdm09 virus aggravates the course of premorbid acute cardiomyopathy in Wistar rats, causing pronounced dysregulation of endothelial factor expression and vasomotor activity impairment of mesenteric arteries.

## 1. Introduction

Influenza is an acute respiratory infection that causes significant illness and death in human populations each year [1]. Influenza A virus (IAV) mainly infects the epithelial cells of conductive airways but can also infect cells of the cardiovascular system including cardiomyocytes, Purkinje cells of the cardiac conduction system, and vascular and cardiac endothelial cells [2,3,4]. Thus, influenza A virus, which was once considered primarily to cause respiratory illness, is now considered as a cardiotropic virus that can cause arrythmias, atrioventricular block, cardiomyopathy, acute cardiac ischemia, myocardial infarction, myocarditis, pericarditis, cardiac tamponade, and congestive heart failure exacerbation [5,6,7,8,9,10]. Persons at higher risk for such influenza-associated complications are patients with acute and chronic cardiovascular and pulmonary disorders [11].

It is known that IAV can infect vascular endothelial cells (EC) which express α(2,3)- and α(2,6)-linked sialic acids [3,12]. Human influenza A(H1N1)pdm09 and A(H3N2) viruses can cause apoptosis, a change in morphology, and modulation of endothelial factors in HUVECs and EA.hy926 ECs [13,14,15]. Experimental infection of Wistar rats revealed that influenza A(H1N1)pdm09 virus caused histopathological changes in pulmonary blood vessel, modulation of endothelial factor expression in the microvascular endothelium of pulmonary vessels, and alteration of vasomotor activity of mesenteric blood vessels in Wistar rats [16,17]. These findings confirm that IAV can lead to endothelial dysfunction (ED).

In turn, ED is considered to cause the onset and progression of cardiovascular diseases, which is consistent with epidemiological data on the correlation between influenza epidemics and the increase in cardiovascular morbidity and mortality (including excess mortality) among hospitalized patients with cardiovascular comorbidities, especially among those 65 years and older [18,19]. In addition, there is a hypothesis that the 1918 pandemic (Spanish flu) led to the subsequent widespread incidence of cardiovascular pathology in the first half of the 20th century [20]. However, the mechanism via which IAV aggravates the course of pre-existing cardiovascular diseases remains not fully understood.

In the present work, we conducted a study on the functional activity of blood vessels in experimental influenza virus infection in Wistar rats with premorbid acute cardiomyopathy.

## 2. Materials and Methods

**Figure 1:**
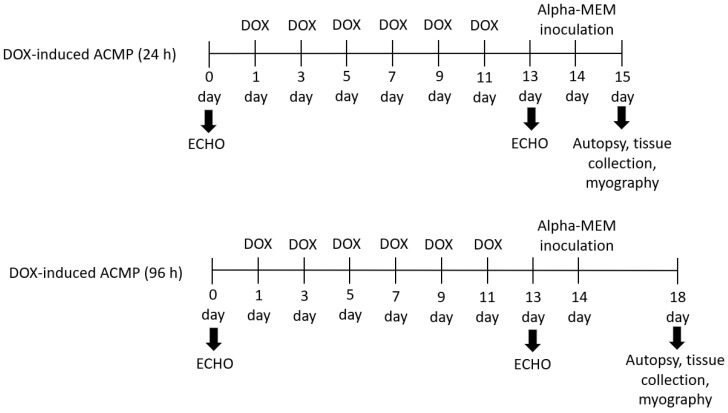

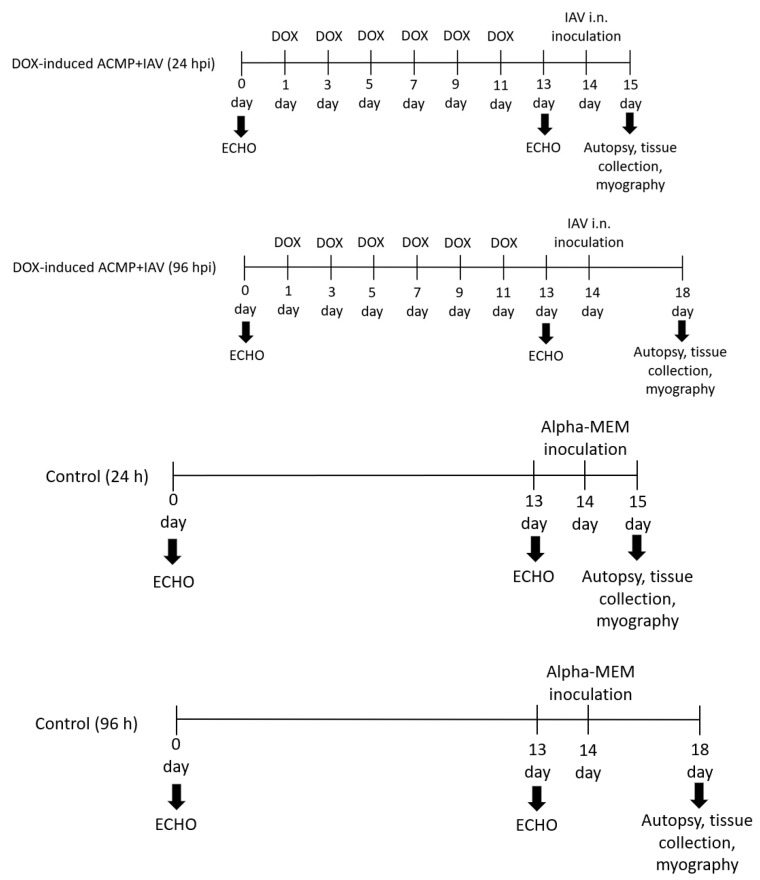
Experimental design. Thirty Wistar rats were randomly placed into six groups (n = 5), including four experimental and two control groups. Each experimental group of rats was treated for 2 weeks with six doses of doxorubicin (DOX) with a cumulative dose 17 mg·kg^−1^. After echocardiographic confirmation of acute cardiomyopathy (DOX-induced ACMP), rats were intranasally inoculated with 0.2 mL of alpha-MEM, while DOX-induced ACMP + IAV rats were infected with 0.2 mL of rat-adapted influenza A/St. Petersburg/48/16 (H1N1)pdm09 virus with autopsy at 24 and 96 h. Control rats were instilled with 0.2 mL of alpha-MEM with autopsy at 24 and 96 h.

Animals. Thirty male Wistar rats (8–10 weeks old) weighing 220–250 g were obtained (Preclinical Translational Research Centre, Saint-Petersburg, Russia) and randomly divided into six groups, including four experimental groups and two control groups (n = 5) (Figure 1).

The rats were housed in shoe-cages with two per cage. Animals were maintained at 22 ± 2 °C and a relative humidity of 50% ± 10% with a 12 h light/dark cycle. All rats received water and food ad libitum. All procedures were approved by the Animal Care and Use Committee of Research Institute of Influenza (protocol No. 031 assigned by 25 February 2021) and were carried out in accordance with the principles of humane treatment of animals, regulated by the requirements of the European Convention (Strasbourg, 1986).

Experimental acute cardiomyopathy. Rats from experimental groups were treated for 2 weeks with six intraperitoneal injection of doxorubicin (DOX) (Veropharm, Russia) with a cumulative dose of 17 mg·kg^−1^. Throughout the experiment, the animals were weighed, and doses were adjusted. To confirm cardiomyopathy development on the next day after last administration of DOX, we performed echocardiography using high-resolution imaging with Visual Sonics Vevo 2100 (Fujifilm, Ontario, Canada). During the study, the following morpho-functional indicators of the left ventricle were recorded:

(1) Anterior wall thickness or IVS;
(2) Posterior wall thickness or LVPW;
(3) Left-ventricular end-diastolic internal diameter or LVIDd;
(4) Left-ventricle end-systolic internal diameter or LVIDs;
(5) Fractional shortening or FS, (LVIDd − LVIDs)/LVIDd × 100.

Virus. Influenza A/St. Petersburg/48/16 H1N1(pdm09) virus was obtained from the Laboratory of Influenza Evolutionary Variability of the Smorodintsev Research Institute of Influenza (St. Petersburg, Russia). The isolate selection was based on clinical studies of Influenza A(H1N1)pdm09 virus, as a causative agent of hemorrhagic pneumonia [21]. The virus was preliminarily adapted through nine successive passages through infected lung homogenates of Wistar rats, as described in [22]. At the last passage, the infectious activity of the influenza virus was 6.6 log EID_50_/mL; the hemagglutination titer of the virus was 1:1024.

Experimental influenza virus infection. Rats from the experimental group after anesthesia with isoflurane were intranasally inoculated with 0.2 mL of rat-adapted influenza A(H1N1)pdm09 virus. Uninfected rats with acute cardiomyopathy and control rats were anesthetized, instilled intranasally with 0.2 mL of alpha-MEM, and necropsied at 24 and 96 h. After necropsy, the lungs, heart, and mesentery were aseptically removed and fixed in 10% neutral buffered formalin for 24–48 h at room temperature with subsequent histological processing.

Tissue lavage and homogenization of collected tissues. After necropsy, the lungs and mesentery were aseptically removed and placed in a pre-weighed petri dish, and the weight of lungs was determined. Then, the tissues were homogenized and centrifuged. The supernatant was collected and stored in aliquots at −70 °C until determination of virus infectious titer.

Determination of influenza virus infectivity titer in embryonated chicken eggs. Viral titer was determined for the lung and mesenteric supernatants using influenza virus propagation in 10–12 day old embryonated chicken eggs and expressed as log EID_50_/mL. Briefly, 10-fold serial dilutions of the supernatant (10^−1^–10^−8^) were prepared in 4.5 mL of PBS (0.01 M phosphate-buffered saline, pH 7.2), and then 0.2 mL from each dilution was used to infect embryonated chicken eggs. After the incubation (36 °C for 48 h), 100 μL of allantoic fluid was taken separately from each embryonated egg and placed in a 96-well plate (Medpolymer, Russian Federation) with the subsequent addition of 100 μL of 0.5% chicken erythrocytes. Viral infectious activity was calculated using the Reed and Muench method [23].

Vasomotor activity of blood vessels. The vascular function of mesenteric arteries was studied using a wire myograph (DMT 620M, Denmark). Immediately after the necropsy of the rat, the mesentery was removed and placed in a Petri dish filled with a cooled Krebs–Henseleit solution of the following composition [mM]: 119 NaCl, 4.7 KCl, 1.17 KH_2_PO_4_, 1.6 CaCl_2_, 1.2 MgSO_4_, 25 NaHCO_3_, 5.5 glucose, and 0.03 EDTA (pH 7.4). After that, three arteries of the third order were isolated from the mesentery.

The blood vessels were mounted in the myograph chamber using two wires with a diameter of 40 μm. Three vessels from each animal in a group were studied (n = 15). After normalization of transmural pressure, the vasocontraction was activated by incubation in Krebs–Henseleit solution with increased potassium ([mM]: 78.2 NaCl, 60 KCl, 1.17 KH_2_PO_4_, 1.6 CaCl_2_, 1.2 MgSO_4_, 25 NaHCO_3_, 5.5 glucose, and 0.03 EDTA) and 10 μM serotonin (5-HT), followed by repeated washing with Krebs–Henseleit solution. To study the vasocontraction, the protocol of cumulative dose-dependent response to serotonin was used. The vessels were incubated in solutions with a phenylephrine (PE) concentration from 10^−7^ to 10^−5^ M. To study endothelium-dependent relaxation, the vessel was preliminarily contracted with PE by 60% of the maximum. Then, incubation with acetylcholine (ACh) was performed according to a scheme similar to the contractile response. The data were recorded using the LabChart 8 software. For the dose-dependent curves obtained, the concentration providing 50% of the maximum response (EC50, μM) and the magnitude of the response at the maximum agonist concentration (Emax, %) were calculated.

Histological analysis. Heart tissues were fixed in 10% neutral buffered formalin for 24–48 h at room temperature. Histological processing was performed using an automatic tissue processor Tissue-Tek VIP 5 (Sakura Finetek, Los Angeles, CA, USA). From paraffin-embedded tissue, block sections with a thickness of 3 μm were made on a rotary microtome Accu-Cut SRM 200 (Sakura, Los Angeles, CA, USA). Sections were dewaxed in xylene, dehydrated in alcohols, and stained with hematoxylin and eosin. Photomicrographs were obtained using a Nikon Eclipse E400 microscope (Nikon, Tokyo, Japan).

Immunohistochemistry. Sections of paraffin-embedded tissues blocks with a thickness of 4–5 μm were placed on poly-L-lysine glass slides (Thermo Fisher Scientific, Waltham, MA, USA). To determine the level of expression of endothelial factors, monoclonal mouse anti-eNOS antibody (Abcam, Bostin, MA, USA, ab76198), polyclonal rabbit anti-PAI-1 antibody (Abcam, Bostin, MA, USA, ab66705), and monoclonal mouse anti-tPA antibody (Novus Biologicals, Littleton, CO, USA, AH54-10) were used. Sections were incubated with antibody at a dilution of 1:200 (for PAI-1, tPA) and 1:500 (for eNOS) for 1 h at room temperature in a humid chamber. To detect the studied endothelial factors expression in the autopsy material, the visualization system Envision Flex was used, which included DAB chromogen (Dako, Glostrup, Denmark) applied for 2–3 min. Then, sections were washed three times, counterstained with Mayer’s hematoxylin, dehydrated in isopropyl alcohol, and cover-slipped.

Morphometric analysis was carried out in Nis-Elements BR 4.40 (Nikon, Tokyo, Japan) with constant settings using blue-channel binarization in automatic mode with a constant threshold [24]. For each endothelial factor, the registration threshold was empirically selected: 0–105 for eNOS, 0–115 for PAI-1, and 0–100 for tPA.

For quantification analysis, three main parameters were chosen:

(1) Measured area—the area of binaries inside the region of interest (ROI).
(2) Sum density—the summed individual optical densities (OD) of each pixel in the ROI; OD was evaluated according to the following formula:
  $$\begin{array}{c} \mathrm{OD}=-\log\;\frac{Pixel\;intensity\;level\;+\;0.5}{Maximum\;intensity\;value}\\ \mathrm{OD}=-\log\;\frac{Pixel\;intensity\;level\;+\;0.5}{Maximum\;intensity\;value} \end{array}$$

(3) Sum intensity—the summed intensity of all pixels of the object under study in the field of view. The intensity was determined in the signal registration range.

Determination of PAI-1 and tPA concentration in rat plasma. Rat plasma was frozen and stored at −20 °C. The concentration of PAI-1 and tPA was analyzed using a commercial kit and standard controls (Abcam, USA). The results of the reaction were taken into account in an enzyme immunoassay analyzer (Anthos, Salzburg, Austria) at a wavelength of 450 nm. The concentration of PAI-1 and tPA in the blood plasma of rats was determined using a standard curve.

*Statistical analysis.* Statistical data processing was performed using nonparametric tests (Wilcoxon, Kruskal–Wallis, Dunnett, one-way analysis of variance (ANOVA), and nonlinear regression) using MS Office Excel 2016 and GraphPad Prism 8. Differences were considered statistically significant for *p* < 0.05. Descriptive statistics such as the standard deviation or standard error of the mean were used to present the obtained data.

## 3. Results

### 3.1. Model of Acute Cardiomyopathy

To confirm the development of acute cardiomyopathy, echocardiography and histological examination of the myocardium were performed.

**Figure 2:**
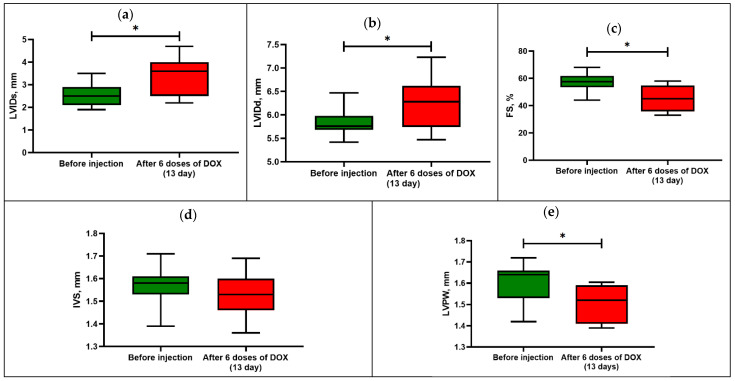
Echocardiography parameters in rats before and after DOX-induced cardiomyopathy: (**a**) left-ventricular end-systolic internal diameter or LVIDs; (**b**) left-ventricular end-diastolic internal diameter or LVIDd; (**c**) fractional shortening or FS; (**d**) anterior wall thickness or IVS; (**e**) posterior wall thickness or LVPW. Box-and-whisker plots ranging from min to max value with the median indicated by horizontal line; * *p* < 0.05 compared with initial values (Wilcoxon sign rank test).

Thus, after the last administration of DOX, the rats showed an increase in the end-systolic and end-diastolic internal diameter of the left ventricle (*p* < 0.05) compared with the initial values (Figure 2).

In this regard, the animals showed a decrease in the fractional shortening by 23% of the initial value (*p* < 0.05), which is one of the indicators of cardiotoxicity development.

There was also a significant difference in the size of the posterior wall thickness of the left ventricle in diastole, while the size of the anterior wall thickness did not differ from the initial values. The action of DOX leads to dilatation of the heart chambers and alteration of the contractile function of the myocardium due to damage with the reduction of functioning cardiomyocytes.

**Figure 3:**
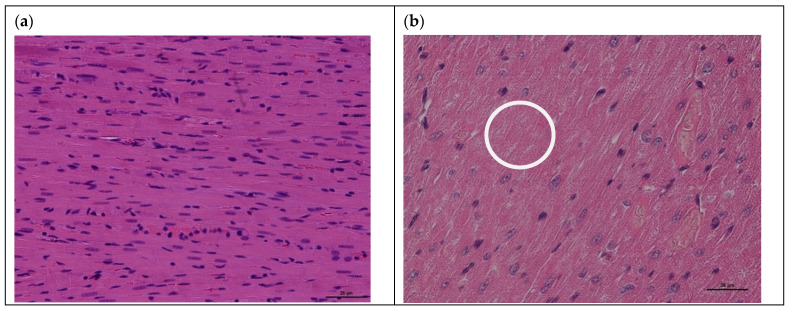
Histological examination (H&E staining) of myocardial tissues of rats after intraperitoneal administration of doxorubicin: (**a**) control rat myocardium; (**b**) rat myocardium after DOX-induced acute cardiomyopathy with following changes: anisonucleosis (cell nuclei of different sizes), necrosis of cardiomyocytes, and karyolysis (white circle) (magnification ×200 for (**a**,**b**)).

In addition, in myocardial tissues of rats with acute cardiomyopathy, the following histopathological changes were observed compared with the control group: anisonucleosis, necrosis of cardiomyocytes, and karyolysis (Figure 3).

### 3.2. Influenza Virus Infectivity Titer in Pulmonary and Mesenteric Tissues

The viral infectivity titer in pulmonary tissues of infected rats with premorbid acute cardiomyopathy at 24 hpi was 7.2 ± 0.3 log EID_50_/mL, while, at 96 hpi, the viral titer was significantly reduced to 2.6 log ± 0.5 EID_50_/mL (Table 1).

Influenza virus infectivity titer in the mesentery tissues was not found over time (at 24 and 96 hpi). In control rats’ pulmonary and mesenteric tissues, virus infectivity titer was also not registered over time.

### 3.3. Vasomotor Activity of Mesenteric Arteries of Rats with Acute Cardiomyopathy

The vasomotor activity of mesenteric arteries of rats with premorbid acute cardiomyopathy was assessed at 24 and 96 hpi (Table 2).

As controls, we used (1) a group of healthy rats (without acute cardiomyopathy and uninfected), and (2) a group of uninfected rats with acute cardiomyopathy.

As shown in Table 2, the vasomotor activity of mesenteric arteries of rats with premorbid acute cardiomyopathy infected with the RA influenza A(H1N1)pdm09 virus differed significantly from the vasomotor activity in both control groups, i.e., uninfected rats with acute cardiomyopathy and healthy rats.

**Figure 4:**
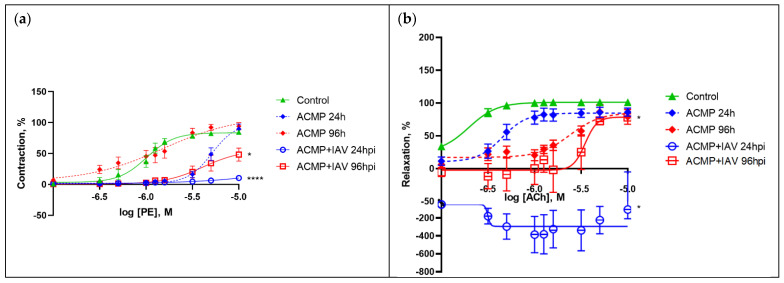
(**a**) Dose-dependent response curves of concentration to phenylephrine (PE) and (**b**) acetylcholine (ACh). Values represent the mean ± SEM from 15 arteries of five rats in every experimental group (n = 30 for control group); * *p* < 0.05, **** *p* < 0.0001 versus control group (Dunnett’s test). Note: Control—healthy rats inoculated with alpha-MEM at 24 and 96 h (combined); ACMP—uninfected rats with premorbid acute cardiomyopathy; ACMP + IAV—rats with premorbid acute cardiomyopathy following infection with influenza A(H1N1)pdm09 virus.

The results of the cumulative dose-dependent response of mesenteric arteries of rats with acute cardiomyopathy to vasoconstriction and a vasodilatation are shown in Figure 4.

Thus, the response of mesenteric arteries of rats with acute cardiomyopathy to the vasoconstrictor at 24 hpi at maximum concentrations (−5 log M) was significantly attenuated by 74.28% compared with healthy rats (*p* < 0.0001) and by 79.16% (additionally by 4.88%) compared with uninfected rats with acute cardiomyopathy (*p* < 0.0001); it was also reduced at 96 hpi by 36.46% compared with healthy rats (*p* < 0.05) and by 45.82% (additionally by 9.36%) compared with uninfected rats with acute cardiomyopathy (*p* < 0.05).

Changes in the response of mesenteric arteries of infected rats with acute cardiomyopathy to the vasodilator were more pronounced, especially at moderate concentrations (−6 log M). Thus, the response of the arteries of rats with acute cardiomyopathy to the vasodilator at 24 hpi at medium concentrations was drastically reduced by 487% compared with the response in healthy rats (*p* < 0.05) and by 464% compared with the response in uninfected rats with acute cardiomyopathy (*p* < 0.05); it was also reduced at 96 hpi by 96% compared with the response in healthy rats (*p* < 0.05), but did not differ from the response in uninfected rats with acute cardiomyopathy.

Thus, influenza A(H1N1)pdm09 virus reduced to various degrees the maximal and dose-dependent responses in the mesenteric arteries of rats with acute cardiomyopathy in response to the vasoconstrictor and vasodilator, at both 24 and 96 hpi.

### 3.4. Expression of eNOS in Mesenteric Blood Vessels of Infected Rats with Premorbid Acute Cardiomyopathy

**Figure 5:**
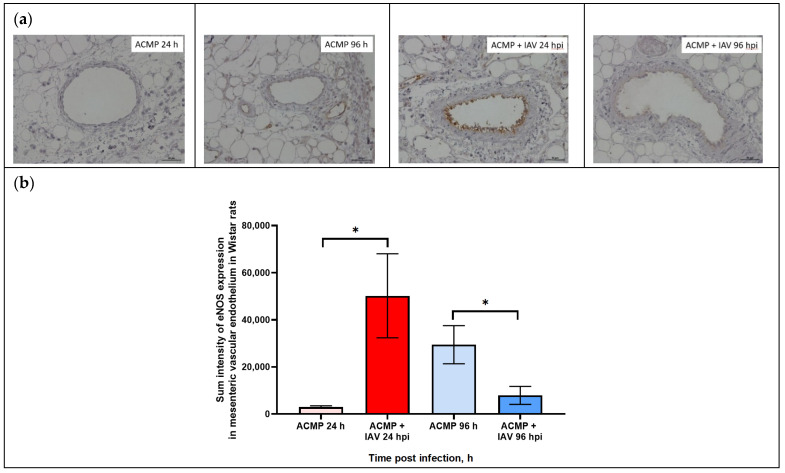
(**a**) eNOS expression in mesenteric vascular endothelium of rats with acute cardiomyopathy (ACMP) and rats with acute cardiomyopathy infected with RA influenza A(H1N1)pdm09 virus using anti-eNOS murine mAbs at 24 and 96 h; magnification ×400, DAB chromogen staining. (**b**) eNOS sum intensity in mesenteric vascular endothelium of rats with acute cardiomyopathy infected with RA influenza A(H1N1)pdm09 virus. Values represent the mean ± standard deviation from 15 blood vessels of five rats in every group. * *p* < 0.05 versus uninfected rats with acute cardiomyopathy (Kruskal–Wallis test).

As shown in Figure 5a,b, eNOS expression in the mesenteric vascular endothelium of uninfected rats with premorbid cardiomyopathy was minimal at 24 hpi, whereas it slightly increased at 96 hpi.

The expression of eNOS in the endothelium of mesenteric blood vessels of rats with acute cardiomyopathy at 24 hpi was significantly increased compared to uninfected rats. In turn, eNOS expression in the mesenteric vascular endothelium of blood of rats with acute cardiomyopathy at 96 hpi was significantly reduced compared with uninfected rats with acute cardiomyopathy.

For quantification of endothelial factor expression, including eNOS, in mesenteric vascular endothelium of rats, three main parameters were determined (Table 3).

As shown in Table 3, the maximal level of signal parameters in endothelial cells was registered in mesenteric blood vessels of rats with acute cardiomyopathy at 24 hpi, while the minimal level was found in uninfected rats with acute cardiomyopathy.

The expression of eNOS expression in the mesenteric vascular endothelium was evaluated by sum intensity parameter.

As shown in Table 3, eNOS expression in the mesenteric vascular endothelium of rats with premorbid acute cardiomyopathy significantly increased 17-fold (50,203.6 ± 17,835.56) at 24 hpi (*p* < 0.05) compared with uninfected rats with premorbid acute cardiomyopathy (2948.7 ± 523.2), but decreased 3.7-fold (7914.0 ± 3819.2) at 96 hpi (*p* < 0.05) compared with uninfected rats with acute cardiomyopathy (29,465.2 ± 8222.41).

### 3.5. Expression of PAI-1 in Mesenteric Blood Vessels of Infected Rats with Premorbid Acute Cardiomyopathy

**Figure 6:**
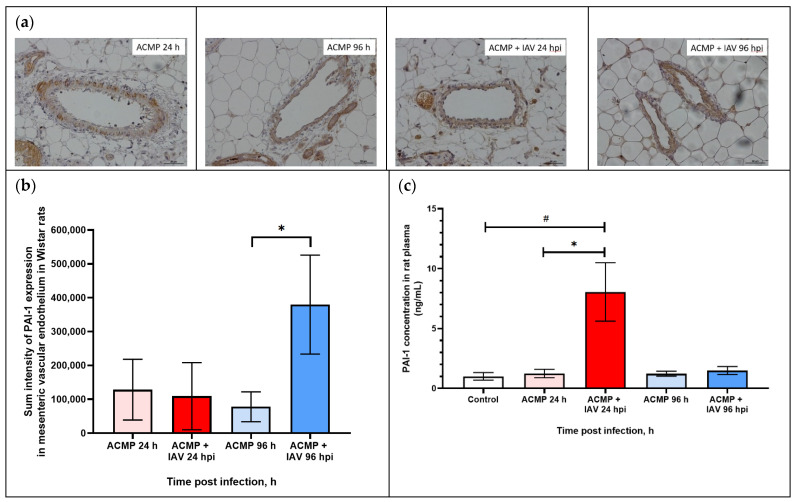
(**a**) PAI-1 expression in mesenteric vascular endothelium and in plasma of rats with acute cardiomyopathy (ACMP) at 24 and 96 h and of rats with acute cardiomyopathy infected with RA influenza A(H1N1)pdm09 virus using anti-PAI-1 rabbit polyclonal Ab; magnification ×400, DAB chromogen staining. (**b**) PAI-1 sum intensity in mesenteric vascular endothelium of rats with acute cardiomyopathy infected with RA influenza A(H1N1)pdm09 virus. (**c**) PAI-1 concentration in blood plasma of rats with acute cardiomyopathy infected with RA influenza A(H1N1)pdm09 virus. Values represent the mean ± standard deviation from 15 blood vessels of five rats in every group (**a**,**b**), and blood plasma of five rats in every group (**c**). * *p* < 0.05 versus uninfected rats with acute cardiomyopathy, ^#^ *p* < 0.05 versus control rats (Kruskal–Wallis test).

As shown in Figure 6a,b, PAI-1 expression in the mesenteric vascular endothelium of uninfected rats and those infected with RA influenza A(H1N1)pdm09 virus with premorbid acute cardiomyopathy was visually on the same level.

However, the morphometric study revealed an increase in PAI-1 expression in the endothelium of the blood vessels of the mesentery of rats at 96 hpi compared with uninfected rats with acute cardiomyopathy.

As shown in Table 4, the sum intensity of PAI-1 in the mesenteric vascular endothelium of rats with acute cardiomyopathy at 24 hpi (109,155.3 ± 99,012.8) practically did not differ from uninfected rats with acute cardiomyopathy (128,267.4 ± 89,587.6), while, at 96 hpi, PAI-1 expression increased 3.47-fold in infected rats with acute cardiomyopathy (379,813.5 ± 146,152.8) compared with uninfected rats with premorbid acute cardiomyopathy (77,930.8 ± 43,845.6) (*p* < 0.05).

### 3.6. Expression of tPA in Mesenteric Blood Vessels of Infected Rats with Premorbid Acute Cardiomyopathy

**Figure 7:**
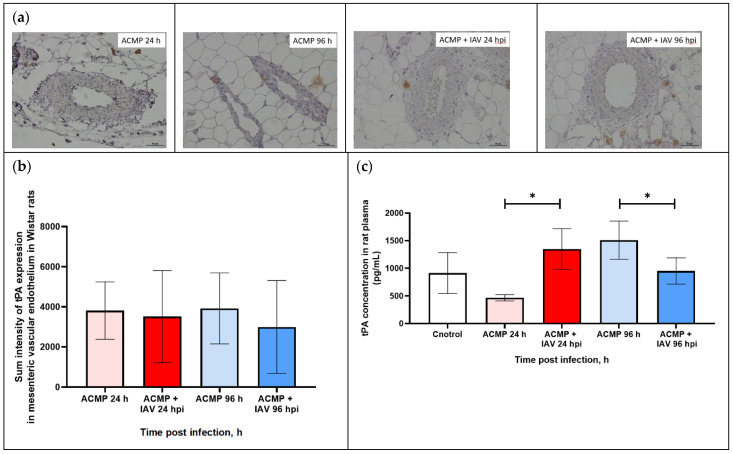
(**a**) tPA expression in mesenteric vascular endothelium and in plasma of rats with acute cardiomyopathy (ACMP) at 24 and 96 h and of rats with acute cardiomyopathy infected with RA influenza A(H1N1)pdm09 virus using anti-tPA murine mAbs; magnification ×400, DAB chromogen staining. (**b**) tPA sum intensity in mesenteric vascular endothelium of rats with acute cardiomyopathy infected with RA influenza A(H1N1)pdm09 virus. (**c**) tPA concentration in blood plasma of rats with acute cardiomyopathy infected with RA influenza A(H1N1)pdm09 virus. Values represent the mean ± standard deviation from 15 blood vessels of five rats in every group (**a**,**b**), and blood plasma of five rats in every group (**c**). * *p* < 0.05 versus uninfected rats with acute cardiomyopathy, ^#^ *p* < 0.05 versus control rats (Kruskal–Wallis test).

As shown in Figure 7a,b, the tPA expression in the mesenteric vascular endothelium of uninfected rats and those infected with RA influenza A(H1N1)pdm09 virus with premorbid acute cardiomyopathy was visually on the same level.

The morphometric analysis also did not reveal any statistical difference in tPA expression (Table 5).

Since PAI-1 and tPA are practically not deposited in endothelial cells and are immediately secreted into the blood after their activation, additional studies were carried out to determine the concentration of PAI-1 and tPA in the blood plasma of rats with premorbid acute cardiomyopathy infected with RA A(H1N1)pdm09 influenza virus.

### 3.7. PAI-1 and tPA Expression in Blood Plasma of Rats with Premorbid Acute Cardiomyopathy

As shown in Figure 6c, the concentration of PAI-1 in infected rats with premorbid cardiomyopathy increased (8.054 ± 2.44 ng/mL) eightfold at 24 hpi compared with healthy rats (control) (1.002 ± 0.31 ng/mL) (*p* < 0.05) and uninfected rats with acute cardiomyopathy (1.243 ± 0.28 ng/mL) (*p* < 0.05), whereas, at 96 hpi, PAI-1 concentration was reduced (1.491 ± 0.33 ng/mL), practically corresponding with control and uninfected rats with acute cardiomyopathy (1.225 ± 0.20 ng/mL; *p* > 0.05).

As shown in Figure 7c, tPA concentration in infected rats with premorbid acute cardiomyopathy increased (1348.5 ± 369.4) 2.88-fold at 24 hpi compared with uninfected rats with acute cardiomyopathy (467.2 ± 57.2; *p* < 0.05). The concentration of tPA was reduced (951.7 ± 237.3) 1.58-fold at 96 hpi compared with uninfected rats with acute cardiomyopathy (1511.1 ± 345.1 ng/mL; *p* < 0.05).

## 4. Discussion

Influenza A virus (IAV) can infect vascular endothelial cells with inducing endothelial dysfunction (ED), which in turn can be the cause of cardiovascular diseases and hematological disorders [18,25,26]. It is also known that cardiac dysfunction is one of the common complications of severe influenza virus infection since IAV can directly infect cardiomyocytes, Purkinje cells of the cardiac conduction system, and endocardium cells [4].

In this study, experimental influenza virus infection was conducted in mature Wistar rats, which had the following advantages: (1) nonlethal infection in rats; (2) rats are one of the main experimental models for studying various cardiovascular disorders, including acute and chronic cardiomyopathy [27].

Acute cardiomyopathy in rats was induced by doxorubicin (DOX), an anthracycline antibiotic. It is known that this antibiotic has the following mechanism of action: (1) disrupts the synthesis of nucleic acids by intercalation between b.p.; (2) inhibits the activity of DNA topoisomerase type II; (3) causes modification of histones, which reduces the rate of DNA repair; (4) increases the formation of reactive oxygen species, which causes oxidative stress and damage to cell membranes and nucleic acids.

In addition to the antimitotic action, administration of DOX can lead to different adverse events, particularly cardiotoxicity. Thus, in vitro studies have shown that DOX increases the synthesis of reactive oxygen species in cardiomyocytes, which contributes to the development of oxidative stress. However, the main damaging factor is the inhibition of DNA topoisomerase II, which leads to breakage of DNA chains and myocardial cell death [28].

Administration of DOX with a cumulative dose 17–20 mg·kg^−1^ for a short period of time (up to 2 weeks) led to acute cardiomyopathy development in rats, which was characterized by alteration of different morpho-functional indicators of the left ventricle including (1) a decrease in left-ventricular fractional shortening, which correlates with a decrease in ejection fraction, (2) an increase in end-systolic left-ventricular diameter, and (3) an increase in end-diastolic left-ventricular diameter.

In this study, we found that the infectious activity of influenza A(H1N1)pdm09 virus in lung homogenates of rats with acute cardiomyopathy at 24 hpi was higher than in lung homogenates of influenza infected Wistar rats without cardiomyopathy (7.2 vs. 6.6 lg EID_50_/mL, respectively) (*p* < 0.05) [17]. One of the possible reasons for higher viral titers in the lung tissues of rats with DOX-induced cardiomyopathy is the pronounced immunosuppressive activity of DOX.

In mesenteric blood vessels and tissues of infected rats, both with and without premorbid acute cardiomyopathy, viral infectivity titer was not registered over time.

The vasomotor activity of mesenteric arteries of rats with acute cardiomyopathy infected with influenza A(H1N1)pdm09 virus was significantly modified compared with control rats and uninfected rats with acute cardiomyopathy. A significant decrease in the maximal response of mesenteric arteries to both the vasoconstrictor and the vasodilator was observed at 24 and 96 hpi. The fact that A(H1N1)pdm09 virus caused an inverted response of mesenteric arteries to vasodilator (acetylcholine) indicates that acetylcholine causes vasoconstriction instead of vasodilation in rats with premorbid acute cardiomyopathy. Such pathological vasomotor activity has been observed in acute coronary syndrome, atherosclerosis of coronary arteries, hypercholesterolemia, and hypertension [29,30].

It should be noted that a decrease in the intensity of vasodilation to acetylcholine by 30% is considered a mild form of endothelial dysfunction, whereas a decrease by 60% or more is considered a severe form of endothelial dysfunction [31]. Thus, influenza A(H1N1)pdm09 virus significantly aggravates ED in rats with premorbid acute cardiomyopathy.

Analysis of endothelial factor expression was carried out in the endothelium of mesenteric blood vessels. Thus, eNOS expression in rats with premorbid acute cardiomyopathy infected with influenza A(H1N1)pdm09 virus increased 17-fold at 24 hpi and decreased 3.72-fold at 96 hpi (compared with uninfected rats with acute cardiomyopathy). Such a high level of eNOS expression at 24 hpi can be associated with the eNOS uncoupling. In this pathological state, eNOS, while continuing to receive electrons from NADP, supplies them to another substrate, molecular oxygen, resulting in the formation of highly active forms of oxygen (in particular, superoxide), which can further cause oxidative stress and increase endothelial dysfunction [32]. A further significant decrease in eNOS expression at 96 hpi indicated the development of systemic endothelial dysfunction and probably indicated the terminal stage of acute heart failure. This conclusion is consistent with the fact that rats with DOX-induced acute cardiomyopathy were viable for 10–14 days; however, after infection with the influenza A(H1N1)pdm09 virus, rats exhibited increased mortality, mainly at 5–7 days post infection.

In addition to eNOS modulation, this study revealed changes in expression level and plasma concentration of two other endothelial factors, tPA and PAI-1. Thus, the expression of PAI-1 in the mesenteric endothelium of infected rats with acute cardiomyopathy increased 3.47-fold at 96 hpi compared with uninfected rats with acute cardiomyopathy. In turn, the concentration of PAI-1 in the blood plasma of infected rats with premorbid acute cardiomyopathy increased 6.43-fold at 24 hpi compared with uninfected rats with acute cardiomyopathy. The concentration of tPA in the blood plasma of infected rats with premorbid acute cardiomyopathy at 24 h after infection increased 2.7-fold and decreased 1.42-fold at 96 hpi compared with uninfected rats with acute cardiomyopathy.

Changes in the expression of tPA and PAI-1 in the mesenteric vascular endothelium and in their concentration in plasma should be considered in combination. It has been established that tPA, in addition to participating in the process of fibrinolysis, plays a role in determining the virulence of the influenza virus, since plasmin (released from plasminogen by tPA cleavage) is also one of the main enzymes that cleave influenza virus hemagglutinin precursor (HA0) [33]. It has been shown that, in addition to playing an important role in the development of inflammatory responses in viral infections, tPA can function as a cytokine, regulating some cell signaling pathways and activating NF-κB, responsible for the development of inflammatory response [34]. In turn, PAI-1 can reduce virulence of the IAV, by being able to bind and inhibit urokinase plasminogen activator (uPA) and tPA, as well as trypsin [35,36]. PAI-1 plays a key role in various thrombotic conditions; a decrease in PAI-1 activity is associated with the development of hemorrhagic syndrome, while its increase may cause thrombosis [37,38].

Overexpression of PAI-1 in the mesenteric vascular endothelium and increased PAI-1 concentration in blood plasma may not only limit the viral infectious activity in the acute phase of infection but also trigger hypercoagulation.

An increased concentration of tPA in plasma at 24 hpi allows the virus to increase its virulence in pulmonary blood vessels and tissues. The decrease in tPA concentration at 96 hpi was associated with the inhibition of this enzyme by increased expression of PAI-1 in mesenteric blood vessels. The modulation of tPA and PAI-1 concentration in blood plasma may indicate the possible development of disseminated intravascular coagulation, which is observed in severe influenza infections [39].

One of the possible mechanisms of dysregulation of endothelial factor expression is viral mimicry of cellular proteins. Previously, we found that different influenza A(H1N1)pdm09 virus strains exhibit molecular homology among all viral proteins and over 20 hemostatic proteins, including eNOS, PAI-1, and tPA [40]. It is quite likely that fragments of viral proteins with a high degree of homology to endothelial factors disorganize vascular hemostasis. Another potential mechanism of this dysregulation may be explained by cross-reactive antibody formation to these homologous fragments in viral and cellular proteins, which can also provoke autoimmune disease.

Thus, influenza A(H1N1)pdm09 virus aggravates the pathology of blood vessels of rats with premorbid acute cardiomyopathy causing pronounced dysregulation of endothelial factor expression and impairment of the vasomotor activity of mesenteric arteries, which in turn indicates the development of systemic endothelial dysfunction.

The obtained results correlate with clinical data indicating a high incidence of severe influenza in patients with cardiovascular comorbidities, which can be a cause of excessive mortality associated with influenza [41].

In addition, the obtained results can serve as a basis for expanding influenza therapy and including endothelial- and vaso-protective medications in combination with etiotropic therapy. This is especially important for elderly patients with cardiovascular comorbidities.

## Figures and Tables

**Table 1:** Infectious titer of rat-adapted influenza A/St. Petersburg/48/16 (H1N1)pdm09 virus in pulmonary tissue of rats (Mean ± SD). Standard deviations for five repeats are shown (* *p* < 0.05 for control No. 1, ^#^ *p* < 0.05 for control No. 2).

Hours Post Infection	Virus Titer (log EID_50_/mL)			Type of Tissue
	Control No. 1 (Healthy Rats)	Control No. 2 (Uninfected Rats with Premorbid Acute Cardiomyopathy)	Rats with Acute Cardiomyopathy Infected with Influenza A(H1N1)pdm09 Virus	
24	0.0	0.0	7.2 ± 0.3 *^,#^	Lungs
96	0.0	0.0	2.6 ± 0.5 *^,#^	
24	0.0	0.0	0.0	Mesentery
96	0.0	0.0	0.0	

**Table 2:** Vasomotor activity of mesenteric arteries in rats. Maximal response and EC_50_ values of mesenteric arteries to vasoconstrictor phenylephrine (PE) and vasodilator acetylcholine (ACh). Values represent the mean ± SEM from 15 arteries of five rats in every experimental group (n = 30 for control group); * *p* < 0.05, ** *p* < 0.01, *** *p* < 0.001, **** *p* < 0.0001 versus control group (Dunnett’s test).

Agonist	Parameter	Control (Healthy Rats) (n = 10)	ACMP 24 h (n = 5)	ACMP 96 h (n = 5)	ACMP + IAV 24 hpi (n = 5)	ACMP + IAV 96 hpi (n = 5)
PE	log EC_50_, M	−6.00 ± 0.04	−5.29 ± 0.05 ****	−2.86 ± 0.56 **	−5.82 ± 0.18	−5.39 ± 0.18 *
	Emax, %	84.66 ± 1.68	89.54 ± 8.00	10.38 ± 2.21 ****	94.02 ± 5.87	48.20 ± 10.59 *
ACh	log EC_50_, M	−6.71 ± 0.18	−6.35 ± 0.07	−6.50 ± 0.86	−5.62 ± 0.10 ***	−5.46 ± 0.10 ***
	Emax, %	100.98 ± 0.73	86.02 ± 6.18	−50.52 ± 32.59 *	86.50 ± 10.79	78.03 ± 5.41 *

**Table 3:** Level of expression of eNOS in the mesenteric vascular endothelium of uninfected rats with acute cardiomyopathy and infected with RA influenza A(H1N1)pdm09 virus.

Signal Parameter	ACMP		ACMP + IAV	
	Time after Inoculation with α-MEM		Time after Infection	
	24 h (n = 5)	96 h (n = 5)	24 h (n = 5)	96 h (n = 5)
Measured area	2387.0 ± 423.5	23,848.8 ± 6548.6	40,641.0 ± 14,438.2 *	6406.5 ± 3091.7 *
Sum density	1544.5 ± 274.0	15,431.4± 4237.3	26,297.1 ± 9342.4 *	5120.8 ± 2000.5 *
Sum intensity	2948.7 ± 523.2	29,460.4 ± 8089.5	50,203.6 ± 17,835.5 *	7914.0 ± 3819.2 *

Values represent the mean ± standard deviation from 15 blood vessels of five rats in every group. * *p* < 0.05 versus uninfected rats with acute cardiomyopathy, ^*^ *p* < 0.05 versus control rats (Kruskal–Wallis test). Note: ACMP—uninfected rats with premorbid acute cardiomyopathy; ACMP + IAV—rats with premorbid acute cardiomyopathy following infection with influenza A(H1N1)pdm09 virus.

**Table 4:** Level of expression of PAI-1 in mesenteric vascular endothelium of uninfected rats with acute cardiomyopathy and infected with RA influenza A(H1N1)pdm09 virus.

Signal Parameter	ACMP		ACMP + IAV	
	Time after Inoculation with α-MEM		Time after Infection	
	24 h (n = 5)	96 h (n = 5)	24 h (n = 5)	96 h (n = 5)
Measured area	111,592.6 ± 72,523.2	60,786.0 ± 35,494.1	84,595.3 ± 80,153.2	300,052.6 ± 118,314.1 *
Sum density	80,282.4 ± 46,926.8	47,120.9 ± 22,966.7	53,541.3 ± 51,863.8	223,919.8 ± 76,556.2 *
Sum intensity	128,267.4 ± 89,587.6	77,930.8 ± 43,845.6	109,155.3 ± 99,012.8	379,813.5 ± 146,152.8 *

Values represent the mean ± standard deviation from 15 blood vessels of five rats in every group. * *p* < 0.05 versus uninfected rats with acute cardiomyopathy, ^#^ *p* < 0.05 versus control rats (Kruskal–Wallis test). Note: ACMP—uninfected rats with premorbid acute cardiomyopathy; ACMP + IAV—rats with premorbid acute cardiomyopathy following infection with influenza A(H1N1)pdm09 virus.

**Table 5:** Level of expression of tPA in mesenteric vascular endothelium of uninfected rats with acute cardiomyopathy and infected with RA influenza A(H1N1)pdm09 virus.

Signal Parameter	ACMP		ACMP + IAV	
	Time after Inoculation with α-MEM		Time after Infection	
	24 h (n = 5)	96 h (n = 5)	24 h (n = 5)	96 h (n = 5)
Measured area	3202.1 ± 1158.6	3409.9 ± 1432.2	3053.2 ± 1863.7	2514.2 ± 1874.6
Sum density	2354.4 ± 749.7	2418.3 ± 926.7	2008.6 ± 1205.9	3670.7 ± 1213.0
Sum intensity	3812.1 ± 1431.3	3919.5 ± 1769.3	3509.5 ± 2302.3	2993.1 ± 2315.8

Values represent the mean ± standard deviation from 15 blood vessels of five rats in every group. * *p* < 0.05 versus uninfected rats with acute cardiomyopathy, ^#^ *p* < 0.05 versus control rats (Kruskal–Wallis test). Note: ACMP—uninfected rats with premorbid acute cardiomyopathy; ACMP + IAV—rats with premorbid acute cardiomyopathy following infection with influenza A(H1N1)pdm09 virus.

## Data Availability

Not applicable.
